# Elastin-Collagen Based Hydrogels as Model Scaffolds to Induce Three-Dimensional Adipocyte Culture from Adipose Derived Stem Cells

**DOI:** 10.3390/bioengineering7030110

**Published:** 2020-09-12

**Authors:** Kristen Newman, Kendra Clark, Bhuvaneswari Gurumurthy, Pallabi Pal, Amol V. Janorkar

**Affiliations:** Department of Biomedical Materials Science, School of Dentistry, University of Mississippi Medical Center, 2500 N State St, Jackson, MS 39216, USA; knewman@alumni.nd.edu (K.N.); rkendra57@yahoo.com (K.C.); bhuvaneswarigurumurthy@gmail.com (B.G.); pink.pallabi@gmail.com (P.P.)

**Keywords:** human adipose derived stem cells, hydrogel, mechanical properties, spheroids

## Abstract

This study aimed to probe the effect of formulation of scaffolds prepared using collagen and elastin-like polypeptide (ELP) and their resulting physico-chemical and mechanical properties on the adipogenic differentiation of human adipose derived stem cells (hASCs). Six different ELP-collagen scaffolds were prepared by varying the collagen concentration (2 and 6 mg/mL), ELP addition (6 mg/mL), or crosslinking of the scaffolds. FTIR spectroscopy indicated secondary bonding interactions between collagen and ELP, while scanning electron microscopy revealed a porous structure for all scaffolds. Increased collagen concentration, ELP addition, and presence of crosslinking decreased swelling ratio and increased elastic modulus and compressive strength of the scaffolds. The scaffold characteristics influenced cell morphology, wherein the hASCs seeded in the softer, non-crosslinked scaffolds displayed a spread morphology. We determined that stiffer and/or crosslinked elastin-collagen based scaffolds constricted the spreading of hASCs, leading to a spheroid morphology and yielded an enhanced adipogenic differentiation as indicated by Oil Red O staining. Overall, this study underscored the importance of spheroid morphology in adipogenic differentiation, which will allow researchers to create more physiologically-relevant three-dimensional, in vitro culture models.

## 1. Introduction

The need to treat obesity is becoming ever more apparent as the obesity related ailments, such as heart disease and diabetes, continue to rise [1,2]. A calorie-restricted diet accompanied by exercise has been the most common treatment regimen prescribed to control body weight. However, better understanding of the mechanisms at play on a cellular scale in the adipose tissue will help develop treatments that directly target the source of the obesity problem, the fat (adipose) tissue. Large numbers of reconstructive and cosmetic surgeries have also brought focus on adipose tissue engineering. Mature adipocytes contain about 90% lipid in their cytoplasm and the mechanical forces due to aspiration damage these cells [3,4]. In addition to their susceptibility to damage during aspiration, mature adipocytes show limited proliferative capabilities, attributed to their terminally differentiated state [5]. Preadipocytes are the precursor cells committed to the adipocyte lineage. These cells are easy to culture, proliferate in vitro, and therefore, make a better candidate for adipose tissue engineering [4]. Stem cells have the ability to differentiate into various cell types upon receipt of suitable stimuli. Embryonic stem cells (ESCs) and mesenchymal stem cells (MSCs) have been widely investigated for adipose tissue engineering because of their ability to mature to an adipogenic lineage [6,7]. Human adipose derived stem cells (hASCs) have gained popularity in recent times due to their clinical relevancy to the human disease. The hASCs can also produce an in vivo-like extracellular matrix (ECM) pattern when cultured as three-dimensional spheroids [8].

Tissue culture techniques to create three-dimensional adipose culture models come in a wide variety. Previous studies have used coated tissue culture polystyrene surfaces, synthetic polymer-based scaffolds, as well as hydrogels prepared using natural ECM-based and peptide-based polymers [6,7,9,10,11,12,13,14]. Cell culture in hydrogel scaffolds has been shown to more readily mimic the three-dimensional in vivo structure of an adipose tissue making it more advantageous to use than a two-dimensional monolayer culture [9]. The availability of materials to prepare the scaffolds, ease of scaffold formation method, and the ability of the scaffold components to mimic the in vivo microenvironment are all important factors in making the appropriate scaffolds for the in vitro, three-dimensional adipose culture models. Gomillion and Burg have presented an extensive review on this research area [12]. Collagen, laminin, fibronectin, and elastin are the major components of the ECM environment of the adipose tissue, out of which collagen is the most abundant [15]. Type I collagen has been used extensively in vivo for wound healing, hemostasis, and guided bone regeneration and in vitro as scaffolds to culture a variety of primary cells [16,17,18]. Unfortunately, most commercially available ECM materials suffer from batch-to-batch variations and high cost. Peptide-based materials provide an attractive alternative to the ECM-based materials due to their controlled chemical structure (amino acid sequence) and molecular weight as well as their stimuli-responsive self-assembly behavior [13,14]. Elastin-like polypeptides (ELPs) and silk-like polypeptides are two examples of widely-used peptide-based biomaterials [13]. We previously prepared composite hydrogels using type I collagen as a minority component (<30% *w/w*) and ELP as the majority component (>70% *w/w*) [19,20,21]. ELPs are a family of peptide-based natural polymers that can be genetically engineered, which eliminates batch-to-batch variation, and their amino acid sequence is derived from the mammalian elastin [22]. ELPs have been shown to be biocompatible and non-immunogenic and have been used for drug delivery and tissue engineering [23,24,25,26,27].

The effects of hydrogel scaffold chemistry, structure (e.g., porosity), and properties (e.g., modulus) on the ultimate function of the encapsulated cells remains an active area of research. Presence of positively charged amine groups have been shown to induce hASC spheroid formation, which led to an enhanced differentiation along adipogenic and osteogenic lineages [28,29,30]. Porosity and pore size also play an important role in supporting adipogenic differentiation of hASCs [31,32]. Scaffold modulus has been shown to have an effect on cell viability and the ability of cells to proliferate and/or differentiate into osteoblasts, myocytes, and neuronal cells [33,34,35,36]. There also seems to be a correlation between the in vivo microenvironment and optimal in vitro microenvironment to achieve high cell viability and functionality [37,38]. There is evidence that scaffold modulus can play a role in the ability of stem cells to differentiate appropriately into specific cell lineages. For example, soft hydrogels (<5 kPa) support chondrogenic/adipogenic differentiation while stiff hydrogels (>20 kPa) induce osteogenic differentiation [39]. However, how the scaffold modulus contributes to the viability and functionality of adipocytes derived from hASCs has not been extensively investigated. Therefore, this study aimed to probe the effect of the scaffold formulation (concentration of collagen, addition of ELP, and crosslinking) and the resulting scaffold mechanical properties (elastic modulus and compressive strength) on the differentiation of hASCs along the adipogenic lineage.

## 2. Materials and Methods

### 2.1. Scaffold Formation

ELP with a primary sequence (valine-proline-glycine-valine-glycine)_120_ was produced as detailed before [19,20,21]. Type I collagen (rat tail tendon; ~9 mg/mL) was purchased from ThermoFisher Scientific, Waltham, MA. Six types of ELP-collagen composite scaffolds were produced (Table 1) as described previously [21]. The scaffold types included: two collagen concentrations (2 mg/mL or 6 mg/mL; referred to as 2C and 6C scaffolds), addition of 6 mg/mL ELP to 2 mg/mL collagen (3:1 mass ratio; referred to as 2C+E scaffold), or crosslinking using ethyl (dimethylaminopropyl) carbodiimide (EDC) and N-Hydroxysuccinimide (NHS) (referred to as 2C_c, 6C_c, and 2C+E_c scaffolds). EDC and NHS were purchased from Sigma Aldrich, St. Louis, MO, USA. Acellular scaffolds were prepared for physical and mechanical characterization and cellular scaffolds were prepared for biochemical characterization. To prepare one acellular scaffold, the needed ingredients (2 or 6 mg/mL collagen, 6 mg/mL ELP, 1:1 weight ratio of EDC:collagen, 1:1 molar ratio of EDC:NHS, and 10X phosphate buffered saline) were mixed together and the final volume was adjusted to 200 μL by adding deionized water. The cellular scaffolds were prepared similarly, except 40,000 hASCs were added into the mixture per scaffold using a concentrated cell suspension of 1 × 10^6^ cells/mL and 10X DMEM cell culture medium was used instead of the 10X phosphate buffered saline. All solutions were kept on ice during the addition of the ingredients. The mixtures were transferred to 96-well plates and then allowed to gel in a 37 °C incubator with >70% humidity for 18 h. The resulting hydrogel scaffolds were about 6 mm thick and 11 mm in diameter.

### 2.2. Scaffold Characterization

Physical characterizations were performed using attenuated total reflectance Fourier transform infrared (ATR-FTIR) spectroscopy, scanning electron microscopy (SEM), and measurement of swelling ratio as detailed elsewhere [19]. ATR-FTIR was performed using a Spectrum 100 FT-IR spectrophotometer (PerkinElmer, Waltham, MA, USA) on freeze-dried scaffolds (*n* = 3) at 4 cm^−1^ resolution. SEM was performed using a SUPRA 40 scanning electron microscope (Carl Zeiss, Thornwood, NY, USA) on freeze-dried scaffolds sputter-coated with Au/Pd at 5 kV (*n* = 3). All SEM images were captured at 1000× magnification. The working distance depended on the individual specimen thickness and varied between 3–9 mm. The percent swelling ratio for the scaffolds (*n* = 6) were calculated as (wet weight—dry weight) × 100/dry weight. The wet weights represented weights of the hydrated scaffolds after gently wicking excess water for 30 s. The dry weights represented weights of the freeze-dried scaffolds.

Mechanical characterization was performed using uniaxial compression testing on hydrated scaffold samples (*n* = 5) using Sintech 2/G Materials Testing System (MTS, Eden Prairie, MN, USA) as detailed before [19]. A strain rate of 1 mm/min until 50% strain was used on scaffold samples placed in a 48-well tissue culture polystyrene plate in a confined manner at room temperature. Scaffolds were incubated in phosphate buffered saline for 8 days at 37 °C, >70% humidity before testing and were kept in phosphate buffered saline during testing. The MTS Testworks 4.0 software was used to calculate the scaffold compressive strength and modulus.

### 2.3. Cell Culture

The hASCs were isolated from elective liposuction aspirates under a protocol approved by the Institutional Review Board of the University of Mississippi Medical Center (Approval # 2012–0004). The hASCs were seeded in scaffolds (40,000 cells/scaffold) as described in Section 2.1 and differentiated for 3 days in 50:50 DMEM and F12 media with 1 μM dexamethasone, 1 μM indomethacin, 0.5 μM IBMX, and 0.1 U/mL insulin. Subsequently, the differentiated cells were given adipocyte maturation media (50:50 DMEM and F12 media with 10% *v/v* FBS and 0.2 U/mL insulin) for up to 11 days.

### 2.4. Biochemical Characterization

DNA content (Cyquant, ThermoFisher Scientific, Waltham, MA, USA) and Oil Red O (Sigma, St. Louis, MO, USA) assays were preformed according to manufacturers’ protocols (*n* = 3). Cells were imaged using an IX-81 microscope (Olympus, Center Valley, PA, USA). Cells stained with Oil Red O were imaged using a VHX digital microscope (Keyence Corp., Osaka, Japan). For quantitative determination of Oil Red O staining, the stain was extracted using isopropanol and absorbance was measured at 405 nm using ELX-800 absorbance plate reader (Winooski, VT, USA). To measure DNA content, cells were first removed from the scaffolds using a collagenase I solution (Sigma) and then lysed using a Branson Digital Sonifier 450 (Danbury, CT, USA).

### 2.5. Statistical Analysis

The results are reported as mean ±95% confidence interval and are analyzed using ANOVA followed by Games–Howell post hoc test. Results were deemed statistically significant at *p* ≤ 0.05 level.

## 3. Results

**Figure 1:**
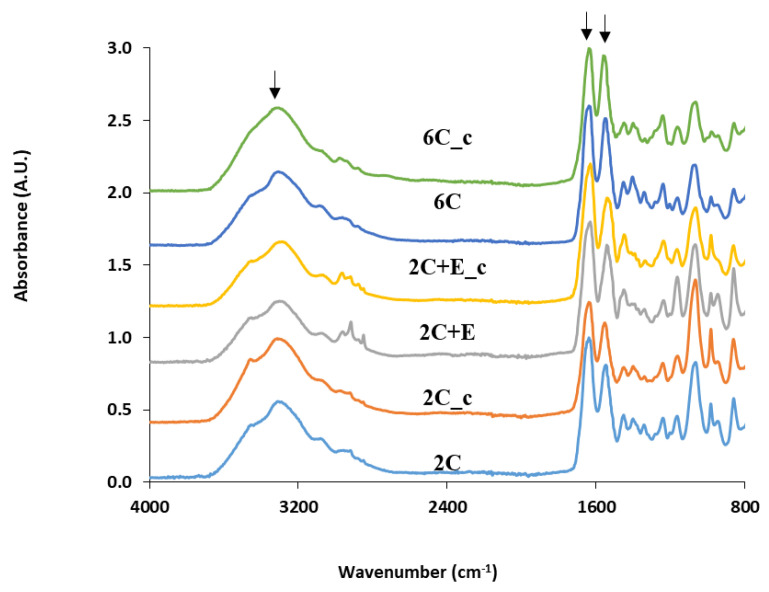
FTIR spectra of the various scaffolds. Arrows indicate peaks around 1640 cm^−1^ (amide-I peak), 1550 cm^−1^ (amide-II peak), and 3300 cm^−1^ (representing the N-H stretching vibrations).

The physico-chemical properties of the scaffolds were analyzed using FTIR spectroscopy, SEM, and swelling ratio. The FTIR spectra (Figure 1) for 2 mg/mL collagen (2C) and 6 mg/mL collagen (6C) scaffolds show the characteristic peaks around 1640 cm^−1^ (amide-I peak representing the C=O stretching vibrations in the amide linkage), 1550 cm^−1^ (amide-II peak representing the combination of C-N stretching and N-H bending vibrations in the amide linkage), and 3300 cm^−1^ (representing the N-H stretching vibrations). The FTIR spectra for the crosslinked 2 mg/mL collagen (2C_c) and 6 mg/mL collagen (6C_c) scaffolds show these characteristic peaks at the same positions. The FTIR spectrum for the 2 mg/mL collagen added with 6 mg/mL ELP (2C+E) scaffold shows the amide-I and amide-II peaks at slightly shifted positions (1630 and 1540 cm^−1^, respectively). Additionally, the 2C+E spectrum shows more prominent peaks around 2850 and 2900 cm^−1^, representing the C-H vibrations in the ELP. The FTIR spectrum for the crosslinked 2C+E (2C+E_c) scaffold shows these characteristic peaks at the same positions.

**Figure 2:**
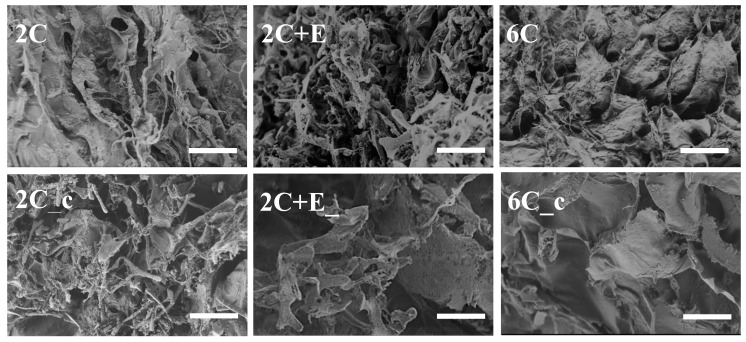
Morphology of the freeze-dried scaffolds assessed using scanning electron microscopy shows a porous structure. All SEM images were captured at 1000× magnification. Scale bar = 50 μm.

The SEM images of all freeze-dried scaffolds showed a porous structure (Figure 2). The SEM images also revealed that the addition of ELP (the 2C+E scaffold) as well as increasing the concentration of collagen to 6 mg/mL (the 6C scaffold) resulted in a denser microstructure compared to the 2 mg/mL collagen (2C) scaffold. The percent swelling ratio of the 2C scaffold was found to be 62 ± 8, indicating the high hydrophilicity of collagen. The percent swelling ratio decreased to 42 ± 6 for the 2C+E scaffold with the addition of the hydrophobic ELP (*p* < 0.05). Increasing the concentration of collagen to 6 mg/mL reduced the percent swelling ratio to 44 ± 5 for the 6C scaffold (*p* < 0.05). Crosslinking also resulted in a reduction in the percent swelling ratio: 41 ± 4 for the 2C_c scaffold (*p* < 0.05 compared to 2C scaffold) and 30 ± 8 for the 6C_c scaffold (*p* < 0.05 compared to 2C and 6C scaffolds). Interestingly, crosslinking of the ELP-containing scaffolds did not further reduce the percent swelling ratio (42 ± 6 for the 2C+E scaffold versus 47 ± 5 for the 2C+E_c scaffold; *p* > 0.05).

**Figure 3:**
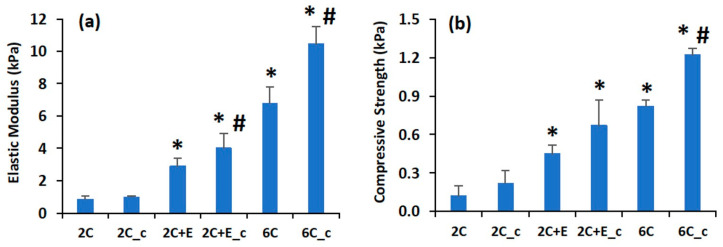
(**a**) Elastic modulus and (**b**) compressive strength of hydrated scaffolds increased with increasing collagen concentration, addition of elastin-like polypeptide (ELP), and/or presence of crosslinking. Error bars indicate 95% C.I. * *p* ≤ 0.05 compared to 2C scaffold. # *p* ≤ 0.05 compared to non-crosslinked scaffold of the same formulation. (**a**,**b**).

The mechanical properties of the hydrated scaffolds were characterized by measuring the elastic modulus and compressive strength (Figure 3). The hydrated 2C scaffold had low elastic modulus (0.8 ± 0.2 kPa) and compressive strength (0.12 ± 0.08 kPa). Addition of ELP and increasing the concentration of collagen to 6 mg/mL led to enhancements in these properties. The 2C+E scaffold displayed an elastic modulus of 2.9 ± 0.5 kPa and compressive strength of 0.45 ± 0.07 kPa, while the 6C scaffold displayed an elastic modulus of 6.8 ± 1.0 kPa and compressive strength of 0.82 ± 0.05 kPa (*p* < 0.05 compared to 2C scaffold). Crosslinking the 2C scaffold did not increase the low elastic modulus and compressive strength values (*p* > 0.05 for 2C_c scaffold compared to 2C scaffold). However, crosslinking the 2C+E and 6C scaffolds further enhanced their elastic modulus and compressive strength (*p* < 0.05). Overall, the 6C_c scaffold displayed the highest elastic modulus of 10.5 ± 1.1 kPa and compressive strength of 1.22 ± 0.05 kPa among the various scaffolds tested (*p* < 0.05).

**Figure 4:**
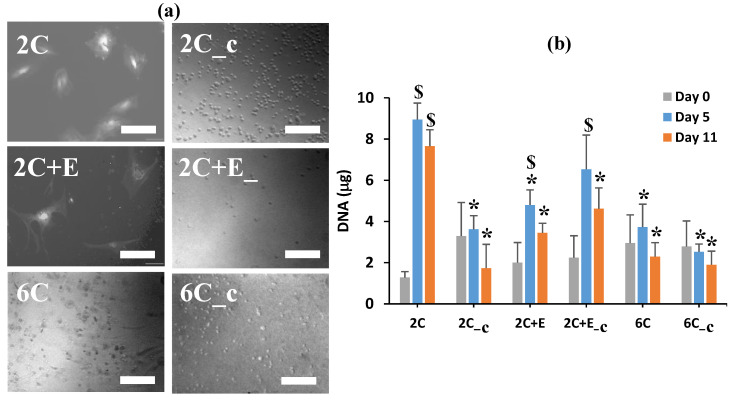
(**a**) Optical microscopy images and (**b**) DNA content of human adipose derived stem cells (hASCs) encapsulated in the various scaffolds. Scale bar = 200 μm. Error bars indicate 95% C.I. * *p* ≤ 0.05 compared to 2C scaffold. $ *p* ≤ 0.05 compared to the DNA content on Day 0 for the scaffold of the same formulation.

Optical microscopy was performed to assess the morphology of the hASCs cultured within the various hydrogel scaffolds. Unfortunately, the high concentration of the collagen used in the 6C and 6C_c scaffolds and the opacity of the 2C+E and 2C+E_c scaffolds due to the addition of ELP made the microscopy imaging challenging. Nevertheless, images could be obtained to determine whether the cells cultured within any particular scaffolds displayed a spread or rounded morphology. The hASCs cultured within the 2C and 2C+E scaffolds displayed a spread morphology; while those cultured within the 2C_c, 2C+E_c, 6C, and 6C_c scaffolds displayed a spheroidal morphology (Figure 4a). A DNA assay was performed to assess proliferation and viability of the incapsulated hASCs in a more quantitative way (Figure 4b). As expected for the same number of cells seeded in each scaffold, the DNA content for all scaffolds was equivalent on day 0 (*p* > 0.05). In the 2C scaffold, the DNA content increased from 1.3 ± 0.3 μg at day 0 to 9.0 ± 0.8 μg at day 5 (*p* < 0.05), which remained constant till day 11 (*p* > 0.05). Similar trend of increasing DNA content was observed for the 2C+E and 2C+E_c scaffolds, albeit to a lesser extent. For the 2C_c, 6C, and 6C_c scaffolds, the DNA content remained constant around 4 μg throughout the 11-day culture period (*p* > 0.05). In general, the DNA assay did not show any detrimental effects of crosslinking. We attribute the increase in the DNA content in the 2C, 2C+E, and 2C+E_c scaffolds to proliferation of hASCs in the initial culture period, while the constant DNA content in the later culture period was likely due to the contact inhibition of cell proliferation in spheroids.

**Figure 5:**
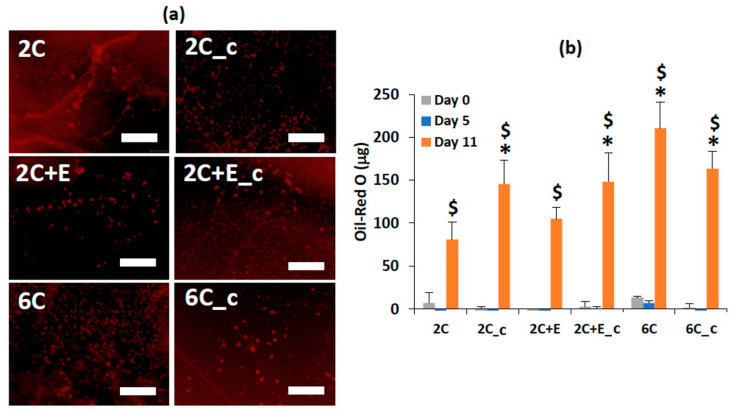
Oil Red O staining (**a**) images and (**b**) quantification to assess the adipogenic differentiation of hASCs encapsulated in the various scaffolds. Scale bar = 200 μm. Error bars indicate 95% C.I. * *p* ≤ 0.05 compared to 2C scaffold. $ *p* ≤ 0.05 compared to Day 0 for the scaffold of the same formulation.

An Oil Red O assay was performed to assess the adipogenic differentiation of hASCs qualitatively (Figure 5a) and quantitatively (Figure 5b). The hASCs seeded in the 2C scaffold showed a significant Oil Red O staining by day 11 (*p* < 0.05), but not by day 5 (*p* > 0.05) compared to day 0. Similar level of Oil Red O staining was observed for the 2C+E scaffold. Interestingly, the 2C_c, 2C+E_c, 6C, and 6C_c scaffolds showed a higher amount of Oil Red O staining by day 11 compared to the 2C and 2C+E scaffolds (*p* < 0.05). Overall, the Oil Red O staining showed maturing adipocytes in all conditions by day 11 but not at day 5, a result consistent with Ramirez-Zacarias et al. [40]. The higher differentiation in the spheroidal aggregates compared to that in cells in the spread morphology was consistent with our previous work showing enhanced adipogenesis in three-dimensional spheroids [10].

## 4. Discussion

Multiple natural materials from mammalian sources have been used as coatings, hydrogels, and sponges to promote differentiation of hASCs and preadipocytes along the adipogenic lineage. Scaffolds have been prepared using ECM-based natural materials including Matrigel [41], type I collagen [42,43], gelatin [7], fibrin [44], as well as decellularized ECM [45,46]. Kelly et al. showed higher neo-adipogenesis in Matrigel scaffolds in contact with in vivo adipose tissue [41], however, Matrigel prepared from the mouse tumor extract is unsuitable for human applications [42]. Type I collagen hydrogel matrix impregnated with fibroblast growth factor-loaded gelatin microspheres was shown to promote adipogenesis in vivo over a six-week implantation period [42]. To develop an alternative to Matrigel, Vashi et al. used these composite scaffolds in a mouse tissue engineering chamber model and demonstrated significant in vivo adipose tissue formation over a six-week period [43]. However, the control Type I collagen matrix hydrogels without the fibroblast growth factor did not induce such extensive adipogenesis [42,43]. More complex ECM-based coating materials obtained from decellularized placenta and adipose tissue have been also been used for two-dimensional monolayer culture of ASCs for up to three weeks [45,46]. The ECM-based natural materials, while considered more in vivo-like, suffer from batch-to-batch variations, shorter shelf-life, and high cost. Therefore, researchers have investigated natural materials from non-mammalian sources such as hyaluronic acid [47,48], alginate [49], silk fibroin [50], and chitosan [28] to promote adipogenesis. Hemmrich et al. used hyaluronic acid gels to encapsulate preadipocytes and showed adipogenic differentiation in vivo in mouse and pig models over a six-week implantation period [47,48], while Halberstadt et al. used RGD-coated alginate scaffolds loaded with ASCs in a sheep model to show adipogenesis over a three-month period [49]. Researchers have also used peptide-based natural materials to promote differentiation of stem cells [13,14,51,52]. Kim et al. improved angiogenesis in a rat myocardial infarction model using a (arginine-alanine-arginine-alanine-aspartic acid-alanine-aspartic acid-alanine)_2_ based hydrogel containing hASCs [51]. Liu et al. used a RAD16 family peptide (RAD16-I) that forms β-sheets in water and self-assembles into scaffolds to support attachment and proliferation of hASCs [52].

Considering that adipocytes grow in volume during their differentiation, mechanically stiff scaffolds used to encapsulate the ASCs and preadipocytes may limit their adipogenesis potential due to their exogenous nature and mechanical constraint. To specifically probe the effect of constraining hASCs during their differentiation along the adipogenic lineage, we formulated several scaffolds with varying physical and mechanical properties using two biocompatible biopolymers, namely, the commercially-available collagen and genetically-engineered ELP. The FTIR spectroscopy showed that all the scaffold types had a similar chemical make-up with respect to amide and amine content (Figure 1) and the shifts in some peaks in the FTIR spectra indicated the secondary (hydrogen) bonding interactions between the collagen and ELP components. The SEM showed that all scaffolds were porous with abundant fibrillar structure (Figure 2). The fibrillar structure may be an artifact of the freeze-drying process. Addition of ELP, increasing the collagen concentration, and crosslinking of the scaffolds decreased the swelling ratio compared to the 2C scaffold, indicating that addition of the ELP provides the 2C+E and 2C+E_c scaffolds a hydrophobic character as established by our previous study [21], while increasing the collagen concentration and crosslinking increases the overall density of the 2C_c, 6C, and 6C_c scaffolds. The hydrogen bonding interactions among the scaffold components, the decreased swelling ratio, and the increased scaffold density led to a systematic enhancement in the elastic modulus and compressive strength of the 2C_c, 2C+E, 2C+E_c, 6C, and 6C_c scaffolds compared to the 2C scaffold (Figure 3). These results corroborate those previously demonstrated by Gurumurthy et al. [21,53]. It is possible that the mechanical properties of the cellular scaffolds are different than the acellular scaffolds characterized in this study. However, since we added the same number of cells in each scaffold, we expect the effects will be similar across the scaffold types.

The modulus values for the various scaffolds prepared in this study (1–10 kPa) were similar to those reported for adipose tissues harvested from human patients [54]. Therefore, we expected a mechanically hospitable environment for the cultured hASCs in our scaffolds as indicated by their successful differentiation along the adipogenic lineage. Nevertheless, the above differences in the scaffold characteristics led to the differences in the hASC morphology as revealed by optical microscopy (Figure 4a). The hASCs seeded in the 2C and 2C+E scaffolds displayed a spread morphology, while those seeded in the 2C_c, 2C+E_c, 6C, and 6C_c scaffolds were shown to form spheroids. These results indicate that the less stiff, less dense, and non-crosslinked scaffolds (2C and 2C+E) were associated with the spread morphology, while increasing stiffness and density (6C) and presence of crosslinking (2C_c, 2C+E_c, and 6C_c) lead to an aggregate morphology. The spread morphology is not representative of the in vivo adipose tissue. The spheroid (aggregate) morphology is the representative in vivo adipose tissue morphology [54]. We posit this to be reason that the hASCs in the spread morphology did not differentiate effectively along the adipogenic lineage compared to the spheroid morphology. The DNA assay also showed differences in proliferation related to the properties of the scaffolds. The 2C and 2C+E scaffolds were shown to have a significantly higher DNA content on day 5 than the other scaffolds (Figure 4b). This increased proliferation was likely due to the spread morphology and possible cell migration that was allowed at the initial culture period by the decreased stiffness and lower density of the 2C and 2C+E scaffolds. The ability to allow cell migration typically reduces contact inhibition, thus leading to increased proliferation. The other scaffolds, however, were stiffer with a higher density and/or restricted the cell migration due to the presence of crosslinking, leading to cell aggregation into a spheroid morphology rather than allowing cell spreading. The contact inhibition of proliferation in such spheroid morphology likely led to the observed lower DNA content, as reported previously by Turner et al. [9,10].

The Oil Red O assay showed differentiation from hASCs to mature adipocytes in all of the scaffolds regardless of composition or properties (Figure 5a). However, there was a difference in the quantitative measurement of the Oil Red O staining based on the properties of the scaffolds (Figure 5b). In the 2C and 2C+E scaffolds, the amounts of Oil Red O stain taken up by the cells were significantly lower than those in the other scaffolds. The higher Oil Red O staining correlated with the spheroid morphology. The increased uptake of Oil Red O correlates to an increase in the adipocyte functionality of the mature cells. Oil Red O is a fat-soluble dye that stains the triglycerides. Previous work by Turner et al. [10], has established that an increased uptake of Oil Red O correlated with an increased production of triglycerides, a functionality of mature adipocytes. This in turn suggests that the hASCs encapsulated in the scaffolds that were stiffer with a higher density and/or restricted the cell migration led to more mature adipocytes through the formation of the spheroid morphology.

In addition to the scaffold mechanical properties, chemical composition of the scaffold materials and the porosity of the scaffolds also affect the adipogenic differentiation of hASCs. We and others have previously shown that changing the surface amine content can affect the size of the hASC spheroids, the stability of the spheroids over the culture period, and their differentiation capability [28,29,30]. While the chemistry experienced by the hASCs in the present study is different for the collagen-only (2C and 6C) scaffolds, the ELP containing (2C+E and 2C+E_c) scaffolds, and the crosslinked (2C_c, 2C+E_c, and 6C_c) scaffolds, our scaffold formation process did not introduce any additional amine groups into our scaffolds. In fact, the number of free amine groups in the crosslinked scaffolds are expected to be less than those present in the non-crosslinked scaffolds as the EDC/NHS crosslinking proceeds by linking free carboxylic acid and amine groups. Considering that cells typically form a two-dimensional monolayer atop collagen and ELP coatings [9,53], formation of spheroids in four out of the six scaffolds studied here (Figure 4) further underscores the influence of mechanical properties on cell morphology. To demonstrate the effect of scaffold porosity and pore size, Oh et al. prepared graded polycaprolactone cylindrical scaffolds with pore size increasing from 90 to 400 μm along the longitudinal direction. Their results indicated 370–400 μm pore size to be optimum for adipogenic differentiation of ASCs [31]. Zubillaga et al. showed successful chondrogenic differentiation of ASCs in chitosan/chitin scaffolds with 95% porosity and 250–500 μm pore size [32]. While all of the scaffolds in the present study were porous (Figure 2), we did not specifically measure their porosity and pore size. It is known that the freeze-drying process can lead to specimens with smaller pore size than present in hydrated specimens. As the specimens were freeze dried in our study for SEM imaging, we suspect that the pore sizes and porosity will be higher in the actual hydrated scaffolds used for the hASC culture.

## 5. Conclusions

Our results indicate that hASC differentiation along the adipogenic lineage is dependent on their morphology, which in turn is governed by the composition, density, and mechanical properties of the scaffolds. We conclude that, while constriction of hASCs may be thought to restrict their ability to differentiate into mature adipocytes, if such a constriction leads to a three-dimensional, in vivo like spheroid morphology, then an enhanced adipogenic hASC differentiation could be expected.

## Figures and Tables

**Table 1:** Selected scaffold compositions.

Name	Collagen	ELP	Crosslinking with EDC/NHS *
2C	2 mg/mL	--	--
2C_c	2 mg/mL	--	Yes
2C+E	2 mg/mL	6 mg/mL	--
2C+E_c	2 mg/mL	6 mg/mL	Yes
6C	6 mg/mL	--	--
6C_c	6 mg/mL	--	Yes

* EDC: ethyl(dimethylaminopropyl) carbodiimide; NHS: N-Hydroxysuccinimide.

## References

[1] Flegal K.M., Carroll M.D., Ogden C.L., Curtin L.R. (2010). Prevalence and trends in obesity among US adults, 1999–2008. J. Am. Med. Assoc..

[2] Pi-Sunyer F.X. (2002). The obesity epidemic: Pathophysiology and consequences of obesity. Obes. Res..

[3] Patrick C.W. (2004). Breast tissue engineering. Annu. Rev. Biomed. Eng..

[4] Katz A.J., Atala A., Lanza R.P. (2002). Mesenchymal cell culture: Adipose tissue. Methods of Tissue Engineering.

[5] Patrick C.W. (2000). Adipose tissue engineering: The future of breast and soft tissue reconstruction following tumor resection. Semin. Surg. Oncol..

[6] Mauney J.R., Nguyen T., Gillen K., Kirker-Head C., Gimble J.M., Kaplan D.L. (2007). Engineering adipose-like tissue in vitro and in vivo utilizing human bone marrow and adipose-derived mesenchymal stem cells with silk fibroin 3D scaffolds. Biomaterials.

[7] Hong L., Peptan I., Clark P., Mao J.J. (2005). *Ex vivo* adipose tissue engineering by human marrow stromal cell seeded gelatin sponge. Ann. Biomed. Eng..

[8] Höfner C., Muhr C., Horder H., Wiesner M., Wittmann K., Lukaszyk D., Radeloff K., Winnefeld M., Becker M., Blunk T. (2020). Human ASC spheroids possess high adipogenic capacity and acquire an adipose tissue-like ECM pattern. Tissue Eng. Part A.

[9] Turner P.A., Weeks C.A., McMurphy A.J., Janorkar A.V. (2014). Spheroid organization kinetics of H35 rat hepatoma model cell system on elastin-like polypeptide–polyethyleneimine copolymer substrates. J. Biomed. Mater. Res. Part A.

[10] Turner P.A., Harris L.M., Purser C.A., Baker R.C., Janorkar A.V. (2014). A surface-tethered spheroid model for functional evaluation of 3T3-L1 adipocytes. Biotechol. Bioeng..

[11] Teong B., Wu S.C., Chang C.M., Chen J.W., Chen H.T., Chen C.H., Chang J.K., Ho M.L. (2017). The stiffness of a crosslinked hyaluronan hydrogel affects its chondro-induction activity on hADSCs. J. Biomed. Mater. Res. Part B.

[12] Gomillion C.T., Burg K.J.L. (2006). Stem cells and adipose tissue engineering. Biomaterials.

[13] Chow D., Nunalee M.L., Lim D.W., Simnick A.J., Chilkoti A. (2008). Peptide-based biopolymers in biomedicine and biotechnology. Mater. Sci. Eng. R Rep..

[14] Hellmund K.S., Koksch B. (2019). Self-Assembling peptides as extracellular matrix mimics to influence stem cell’s fate. Front. Chem..

[15] Divoux A., Tordjman J., Lacasa D., Veyrie N., Hugol D., Aissat A., Basdevant A., Guerre-Millo M., Poitou C., Zucker J.D. (2010). Fibrosis in human adipose tissue: Composition, distribution, and link with lipid metabolism and fat mass loss. Diabetes.

[16] Liu X., Zheng C., Luo X., Wang X., Jiang H. (2019). Recent advances of collagen-based biomaterials: Multi-hierarchical structure, modification and biomedical applications. Mater. Sci. Eng. C.

[17] Lin K., Zhang D., Macedo M.H., Cui W., Sarmento B., Shen G. (2019). Advanced collagen-based biomaterials for regenerative biomedicine. Adv. Healthcare Mater..

[18] Parenteau-Bareil R., Gauvin R., Berthod F. (2010). Collagen-based biomaterials for tissue engineering applications. Materials.

[19] Pal P., Nguyen Q., Hollis A., Marquart M.E., Janorkar A.V. (2019). Drug-loaded elastin-like polypeptide-collagen hydrogels with high modulus for bone tissue engineering. Macromol. Biosci..

[20] Clark K., Janorkar A.V. (2018). Milieu for endothelial differentiation of human adipose-derived stem cells. Bioengineering.

[21] Gurumurthy B., Griggs J.A., Janorkar A.V. (2018). Optimization of collagen-elastin-like polypeptide composite tissue engineering scaffolds using response surface methodology. J. Mech. Behav. Biomed. Mater..

[22] Miao M., Bellingham C.M., Stahl R.J., Sitarz E.E., Lane C.J., Keeley F.W. (2003). Sequence and structure determinants for the self-aggregation of recombinant polypeptides modeled after human elastin. J. Biol. Chem..

[23] Srokowski E.M., Woodhouse K.A. (2008). Development and characterization of novel cross-linked bio-elastomeric materials. J. Biomater. Sci. Polym. Ed..

[24] Liu J.C., Tirrell D.A. (2008). Cell response to RGD density in cross-linked artificial extracellular matrix protein films. Biomacromolecules.

[25] Lee J., Macosko C.W., Urry D.W. (2001). Elastomeric polypentapeptides crosslinked into matrices and fibers. Biomacromolecules.

[26] Huang L., McMillan R.A., Apkarian R.P., Pourdeyhimi B., Conticello V.P., Chaikof E.L. (2000). Generation of synthetic elastin-mimetic small diameter fibers and fiber networks. Macromolecules.

[27] Arias F.J., Reboto V., Martin S.M., Lopez I., Rodríguez-Cabello J.C. (2006). Tailored recombinant elastin-like polymers for advanced biomedical and nano(bio)technological applications. Biotechnol. Lett..

[28] Cheng N.C., Wang S., Young T.H. (2012). The influence of spheroid formation of human adipose-derived stem cells on chitosan films on stemness and differentiation capabilities. Biomaterials.

[29] Gurumurthy B., Bierdeman P.C., Janorkar A.V. (2017). Spheroid model for functional osteogenic evaluation of human adipose derived stem cells. J. Biomed. Mater. Res. Part A.

[30] Fitzgerald S.J., Cobb J.S., Janorkar A.V. (2020). Comparison of the formation, adipogenic maturation, and retention of human adipose-derived stem cell spheroids in scaffold-free culture techniques. J. Biomed. Mater. Res. B Appl. Biomater..

[31] Oh S.H., Kim T.H., Im G.I., Lee J.H. (2010). Investigation of pore size effect on chondrogenic differentiation of adipose stem cells using a pore size gradient scaffold. Biomacromolecules.

[32] Zubillaga V., Alonso-Varona A., Fernandes S.C.M., Salaberria A.M., Palomares T. (2020). Adipose-derived mesenchymal stem cell chondrospheroids cultured in hypoxia and a 3D porous chitosan/chitin nanocrystal scaffold as a platform for cartilage tissue engineering. Int. J. Mol. Sci..

[33] Langer R., Tirrell D.A. (2004). Designing materials for biology and medicine. Nature.

[34] Lutolf M., Hubbell J.S. (2005). Synthetic biomaterials as instructive extracellular microenvironments for morphogenesis in tissue engineering. Nat. Biotechnol..

[35] Wells R.G. (2008). The role of matrix stiffness in regulating cell behavior. Hepatology.

[36] Cigognini D., Lomas A., Kumar P., Satyam A., English A., Azeem A., Pandit A., Zeugolis D. (2013). Engineering in vitro microenvironments for cell based therapies and drug discovery. Drug. Discov. Today.

[37] Guilak F., Cohen D.M., Estes B.T., Gimble J.M., Liedtke W., Chen C.S. (2009). Control of stem cell fate by physical interactions with the extracellular matrix. Cell Stem Cell.

[38] Reilly G.C., Engler A.J. (2010). Intrinsic extracellular matrix properties regulate stem cell differentiation. J. Biomech..

[39] Jaiswal M.K., Xavier J.R., Carrow J.K., Desai P., Alge D., Gaharwar A.K. (2016). Mechanically stiff nanocomposite hydrogels at ultralow nanoparticle content. ACS Nano.

[40] Ramirez-Zacarias J.L., Castro-Muñozledo F., Kuri-Harcuch W. (1992). Quantitation of adipose conversion and triglycerides by staining intracytoplasmic lipids with Oil red O. Histochemistry.

[41] Kelly J.L., Findlay M.W., Knight K.R., Penington A., Thompson E.W., Messina A., Morrison W.A. (2006). Contact with existing adipose tissue is inductive for adipogenesis in Matrigel. Tissue Eng..

[42] Kimura Y., Ozeki M., Inamoto T., Tabata Y. (2003). Adipose tissue engineering based on human preadipocytes combined with gelatin microspheres containing basic fibroblast growth factor. Biomaterials.

[43] Vashi A.V., Abberton K.M., Thomas G.P., Morrison W.A., O’Connor A.J., Cooper-White J.J., Thompson E.W. (2006). Adipose tissue engineering based on the controlled release of fibroblast growth factor-2 in a collagen matrix. Tissue Eng..

[44] Torio-Padron N., Baerlecken N., Momeni A., Stark G.B., Borges J. (2007). Engineering of adipose tissue by injection of human preadipocytes in fibrin. Aesthet. Plast. Surg..

[45] Flynn L., Semple J.L., Woodhouse K.A. (2006). Decellularized placental matrices for adipose tissue engineering. J. Biomed. Mater. Res. Part A.

[46] Mohiuddin O.A., O’Donnell B.T., Poche J.N., Iftikhar R., Wise R.M., Motherwell J.M., Campbell B., Savkovic S.D., Bunnell B.A., Hayes D.J. (2019). Human adipose-derived hydrogel characterization based on in vitro ASC biocompatibility and differentiation. Stem Cells Int..

[47] Hemmrich K., von Heimburg D., Rendchen R., Di Bartolo C., Milella E., Pallua N. (2005). Implantation of preadipocyte-loaded hyaluronic acid based scaffolds into nude mice to evaluate potential for soft tissue engineering. Biomaterials.

[48] Hemmrich K., Van de Sijpe K., Rhodes N.P., Hunt J.A., Di Bartolo C., Pallua N., Blondeel P., von Heimburg D. (2008). autologous in vivo adipose tissue engineering in hyaluronan-based gels—A pilot study. J. Surg. Res..

[49] Halberstadt C., Austin C., Rowley J., Culberson C., Loebsack A., Wyatt S., Coleman S., Blacksten L., Burg K., Mooney D. (2002). A hydrogel material for plastic and reconstructive applications injected into the subcutaneous space of a sheep. Tissue Eng..

[50] Choi J.H., Bellas E., Vunjak-Novakovic G., Kaplan D.L. (2011). Adipogenic differentiation of human adipose-derived stem cells on 3D silk scaffolds. Methods Mol. Biol..

[51] Kim J.H., Park Y., Jung Y., Kim S.H., Kim S.H. (2017). Combinatorial therapy with three-dimensionally cultured adipose-derived stromal cells and self-assembling peptides to enhance angiogenesis and preserve cardiac function in infarcted hearts. J. Tissue Eng. Regen. Med..

[52] Liu X., Wang X., Wang X., Ren H., He J., Qiao L., Cui F. (2013). Functionalized self-assembling peptide nanofiber hydrogels mimic stem cell niche to control human adipose stem cell behavior in vitro. Acta Biomater..

[53] Gurumurthy B., Pal P., Griggs J.A., Janorkar A.V. (2020). Optimization of collagen-elastin-like polypeptide-bioglass scaffold composition for osteogenic differentiation of adipose-derived stem cells. Materialia.

[54] Alkhouli N., Mansfield J., Green E., Bell J., Knight B., Liversedge N., Tham J.C., Welbourn R., Shore A.C., Kos K. (2013). The mechanical properties of human adipose tissues and their relationships to the structure and composition of the extracellular matrix. Am. J. Physiol. Endocrinol. Metab..

